# Synergetic Inactivation Mechanism of Protocatechuic Acid and High Hydrostatic Pressure against *Escherichia coli* O157:H7

**DOI:** 10.3390/foods10123053

**Published:** 2021-12-08

**Authors:** Jingyi Hao, Yuqing Lei, Zhilin Gan, Wanbin Zhao, Junyan Shi, Chengli Jia, Aidong Sun

**Affiliations:** 1 College of Biological Sciences and Biotechnology, Beijing Forestry University, No. 35 Qinghua East Road, Haidian District, Beijing 100083, China; jyhao0529@126.com (J.H.); reveriecity@foxmail.com (Y.L.); ganzhilin@bjfu.edu.cn (Z.G.); 13161526700@163.com (W.Z.); shijunyan0130@126.com (J.S.); Adler_Jia@163.com (C.J.); 2 Beijing Key Laboratory of Food Processing and Safety in Forestry, No. 35 Qinghua East Road, Haidian District, Beijing 100083, China

**Keywords:** high hydrostatic pressure, protocatechuic acid, *Escherichia coli* O157:H7, synergetic bactericidal, mechanism

## Abstract

With the wide application of high hydrostatic pressure (HHP) technology in the food industry, safety issues regarding food products, resulting in potential food safety hazards, have arisen. To address such problems, this study explored the synergetic bactericidal effects and mechanisms of protocatechuic acid (PCA) and HHP against *Escherichia coli* O157:H7. At greater than 200 MPa, PCA (1.25 mg/mL for 60 min) plus HHP treatments had significant synergetic bactericidal effects that positively correlated with pressure. After a combined treatment at 500 MPa for 5 min, an approximate 9.0 log CFU/mL colony decline occurred, whereas the individual HHP and PCA treatments caused 4.48 and 1.06 log CFU/mL colony decreases, respectively. Mechanistically, membrane integrity and morphology were damaged, and the permeability increased when *E. coli* O157: H7 was exposed to the synergetic stress of PCA plus HHP. Inside cells, the synergetic treatment additionally targeted the activities of enzymes such as superoxide dismutase, catalase and ATPase, which were inhibited significantly (*p* ≤ 0.05) when exposed to high pressure. Moreover, an analysis of circular dichroism spectra indicated that the synergetic treatment caused a change in DNA structure, which was expressed as the redshift of the characteristic absorption peak. Thus, the synergetic treatment of PCA plus HHP may be used as a decontamination method owing to the good bactericidal effects on multiple targets.

## 1. Introduction

Outbreaks caused by foodborne pathogens have received public and media attention because of their risks to consumers. *Escherichia coli* O157:H7 is a harmful and common foodborne pathogenic bacteria that causes life-threatening infectious diseases [1]. The Centers for Disease Control and Prevention estimate that O157:H7 results in over 265,000 medical cases annually in the USA, which have not only medical costs but also economic consequences [2]. *Escherichia coli* O157:H7 adapts to environmental and processing stresses [3], which might present potential dangers to food safety. Although traditional thermal treatments have decent bactericidal effects, temperature-sensitive nutrients in food, such as anthocyanins, are destroyed during these processes [4]. Therefore, it is necessary to find a method that can both protect temperature-sensitive nutrients and ensure food safety.

With the increasing consumer demand for fresh food, having an improved nutritional quality, several non-thermal treatments have been introduced [5]. Among them, high hydrostatic pressure (HHP) technology has been widely applied to the food industry already, owing to the advantages of low energy consumption, limited contamination and environmental friendliness [6,7]. Among the non-thermal processing techniques, HHP is the only technology that has been successfully commercialized in the last decade [8]. HHP treatments result in changes in membranes, including membrane potential, membrane fluidity, membrane permeability, and even the membrane-related genes [9,10]. Moreover, HHP might result in morphological changes, intracellular content loss and DNA and protein denaturation [11,12]. However, the bacteria may be induced by HHP into sub-lethal conditions, which could be repaired in a proper environment [13,14]. Thus, preservation using a single HHP technology does not ensure the complete safety of food products.

Protocatechuic acid (PCA, 3,4-dihydroxybenzoic acid) is a water-soluble natural product that exists widely in edible plants, fruits and vegetables [15]. Its antibacterial actions against foodborne pathogens, such as *Staphylococcus aureus*, *Escherichia coli* and *Listeria monocytogenes*, might be attributed to the disruption of cell membranes and the subsequent increase in membrane permeability [16,17,18]. Bernal-Mercado et al. also demonstrated that PCA prevents *E. coli* adhesion and biofilm formation [19]. Additionally, PCA might target the intracellular environment and the oxidative stress system [16,20]. Consequently, a combination of PCA and HHP treatments might be an effective way to achieve the desired level of microbial inactivation. The combination of HHP and dissolved CO_2_ has strong bactericidal effects on *Staphylococcus aureus* and *E. coli* [21]. Nassau et al. [22] also found that the combination of HHP and endolysins demonstrates a more than additive effect, which indicated the feasibility of a synergetic bactericidal method. However, no studies have focused on the synergetic bactericidal effects and mechanisms of PCA and HHP treatments.

The objectives of this study were to investigate comprehensively the bactericidal effects and mechanisms, including those related to membranes, intracellular macromolecules, enzyme activities and morphology. The current study provides a way to offset HHP’s bactericidal effect-related deficiency, and explores PCA’s potential as a food additive.

## 2. Materials and Methods

### 2.1. Reagents and Culture Mediums

Protocatechuic acid (PCA, purity ≥ 99.5%) and propidium iodide (PI, 1 mg/mL) were purchased from Solarbio life sciences, Co., Ltd. (Beijing, China). Ampicillin (1 mg/mL) and glutaraldehyde fixative (2.5%) were purchased from BioDee Biotechnology Co., Ltd. (Beijing, China). The Bradford kit, potassium (K^+^) assay kit, magnesium (Mg^2+^) assay kit, superoxide dismutase (SOD) assay kit (WST-1 method), catalase (CAT) assay kit (visible light), and minim adenosine triphosphate enzyme (ATPase) test kit were all purchased from Jiancheng Bioengineering Institute (Nanjing, China). The bis-(1,3-dibutylbarbituric acid) trimethine oxonol (DiBAC (4)_3_) dye and phosphate buffer (PBS, pH 7.2–7.4) were purchased from UE Everbright Incorporation (Suzhou, China) and Biotopped Co., Ltd. (Beijing, China), respectively.

The nutrient broth culture medium (NB) and tryptose soya broth culture medium (TSB) was purchased from Aoboxing Biotech Co., Ltd. (Beijing, China). The tryptose soya ager (TSA) was purchased from Qingdao Hope Bio-Technology Co., Ltd. (Qingdao, China).

### 2.2. Strain Cultivation

The *E. coli* O157:H7 NCTC 12900 strain, was purchased from the China Center of Industrial Culture Collection (Beijing, China) and stored at −20 °C. The strain was activated by incubating in NB at 37 °C for 7 h and then transferred to NB supplemented with 20% agar powder slant cultures and stored at 4 °C. After streak culturing in TSA, a loop of a single colony was then transferred to 50 mL TSB (pH 7.2), incubated in a shaker (TS-100B, Shanghai Tiancheng Experimental Instrument Manufacturing Co., Ltd., Shanghai, China) at 37 °C, 180 rpm for 6 h until the culture reached the middle period of logarithmic phase [23]. The bacterial pellet was harvested by centrifugation at 6000 rpm and 4 °C for 10 min, and then washed three times with PBS to remove the excess medium. The concentration of the final bacterial suspension (in PBS) obtained was approximately 10^8^–10^9^ colony forming units per milliliter (CFU/mL).

### 2.3. Determination of the Protocatechuic Acid (PCA) Treatment Condition

The minimum inhibitory concentration (MIC) of PCA against *E. coli* O157:H7 was determined in accordance with Shi et al. [24] with slight modifications. Briefly, 1 mL PCA solutions of various concentrations (20, 10, 5, 2.5, 1.25 mg/mL) were mixed individually into 1 mL sterile TSA culture medium (cooled to 40–50 °C) to form the final 10, 5, 2.5, 1.25 and 0.625 mg/mL PCA concentrations in a sterile 24-well microplate. After the medium solidified, 2 μL bacterial suspension was added to the center of the medium and then incubated at 37 °C for 24 h. The negative control was the culture medium without PCA (1 mL TSA mixed with 1 mL water), whereas the positive control was the culture medium supplemented with ampicillin. The MIC was defined as the lowest PCA concentration that showed no visible growth of the test bacteria [25]. Afterwards, the pellet was re-suspended in 50 mL PCA solution to 1/8 MIC (0.3125 mg/mL) and then cultured for 15, 30, 45, 60 and 75 min, respectively (120 rpm, 37 °C). Additionally, the pellet was re-suspended to 1/4, 1/2 and 1 MIC (0.625, 1.25 and 2.5 mg/mL) under the same conditions. Then, 100 μL of each treated bacterial solution was spread on the TSA medium and incubated at 37 °C for 12 h. The bactericidal effects of these treatments were determined using the colony counting method and expressed using a logarithm of the colony number (log CFU/mL).

### 2.4. Synergetic Treatment

The study had two experimental groups, the HHP and synergetic (PCA + HHP) treatment groups. In the HHP group, 10 mL bacterial suspension was packaged per aseptic sealed bag, and then, the bags were treated independently with 0, 100, 200, 300, 400 and 500 MPa for 5 min at 25 °C using HHP equipment (Shanghai Litu Ultra-High Voltage Equipment Co., Ltd., Shanghai, China). The rise (approximately 2–3 min) and relief (approximately 1–2 s) times were not included in the processing time. In the PCA + HHP group, the samples were pre-treated with a 1/2-MIC PCA aqueous solution (1.25 mg/mL, pH 3.27, filtered with 0.22-μm microporous membrane-water system). The pellets were each re-suspended in 10 mL 1/2-MIC PCA solution, and then cultured at 37 °C for 60 min at 120 rpm. The bacterial suspension after the PCA treatment was then treated under the same conditions as the HHP group [23]. For the 0 MPa treatment of the PCA + HHP group, cells treated with 1/2-MIC PCA, were then placed in the HHP equipment for 5 min at room temperature. The bactericidal effects of these two treatments were determined using the bacterial logarithm reduction. The initial concentration of the bacterial suspension was approximately 10^8^–10^9^ CFU/mL.

### 2.5. Determination of Membrane Permeability

Membrane permeability was determined using the PI staining method in accordance with Raffellini et al. [26]. Briefly, 5 µL of PI solution was added to 1 mL of 10-fold diluted bacterial suspension (approximately 10^7^–10^8^ CFU/mL), and then incubated at room temperature for 30 min in the dark. The mixture was washed two times with PBS buffer to remove excess dye by centrifugation at 6000 rpm and 4 °C for 10 min). The pellet was resuspended in 1 mL PBS for flow cytometry detection (FACSCalibur, Becton, Dickinson and Company, Franklin Lakes, NJ, USA). The excitation wavelength was 488 nm, and a total of 50,000 cells were detected.

### 2.6. Determination of the Intracellular Constituent Contents

The bacterial suspensions subjected to different treatment were centrifuged by 6000 rpm and 4 °C for 10 min. Each pellet was re-suspended in PBS and then crushed using an ultrasonic processor (HY92-IIDN, Ningbo Scienta Biotechnology Co., Ltd., Ningbo, China) in an ice bath to obtain the intracellular solution. The concentration of the intracellular protein was determined using a Bradford kit, which involved mixing the intracellular solution and Coomassie brilliant blue stock solution in a ratio of 1:60 to determine the absorbance at 595 nm using an UV-vis spectrophotometer (UV 6100, Shanghai Metash Instruments Co., Ltd., Shanghai, China). The final concentration was determined by comparing and converting the absorbance with that of the standard protein solution (bovine serum albumin, 0.563 mg/mL). The intracellular nucleic acid content was determined at OD_260 nm_ using a UV-vis spectrophotometer in accordance with previous research [27] with modifications. The intracellular K^+^ and Mg^2+^ concentrations were determined using potassium [28] and magnesium assay kits [29], respectively, with slight modifications. For the Mg^2+^ concentration, the samples were mixed 1:4 with the complexation indicator calmagite. After 1 to 2 min of incubation at 37 °C, the absorbance was measured at 540 nm. The K^+^ concentration was obtained by mixing samples at a 1:4 ratio with sodium tetraphenylborate and determining the absorbance at 440 nm. To avoid the influence of K^+^ and Mg^2+^ ions in PBS, each pellet was washed and re-suspended in 0.85% NaCl solution instead of PBS. The initial concentration of the bacterial suspension was approximately 10^8^–10^9^ CFU/mL.

### 2.7. Determination of Membrane Potential

DiBAC(4)_3_ dye was used to determine the change in membrane potential in accordance with Nuding et al. [30]. Briefly, 2 μL of the diluted DiBAC(4)_3_ solution (0.5 mg/mL final concentration) was added to 1 mL 10× diluted bacterial suspension (approximately 10^7^–10^8^ CFU/mL concentration). The mixture was shaken to mix thoroughly, and then incubated at room temperature for 30 min in the dark. Flow cytometry detection was used to observe the fluorescence intensity, and the excitation wavelength was 517 nm.

### 2.8. Determination of Key Enzyme Activities

The activities of intracellular oxidative stress-related SOD and CAT were determined using the SOD and CAT assay kits, respectively [31]. The experiments were conducted as per the instructions, and the absorbance levels were measured at 450 and 405 nm, respectively. Each enzyme activity was expressed as relative enzyme activity, in which A*X* and A0 represented the enzyme activities of samples exposed to different treatments (*X*) and the untreated sample, respectively.

(1) $$\mathrm{Enzyme}\;\mathrm{activity}\%=\frac{AX}{A0}\times 100$$

The intracellular solution, described previously (in 0.85% NaCl solution) was used to determine the Na^+^ K^+^-ATPase, Ca^2+^ Mg^2+^-ATPase and total-ATPase activities using a Minim ATP enzyme test kit. The pellet was suspended in cold sterile water and then ultrasonicated to obtained the enzyme solution. The finally result was measured at 636 nm, and the unit of ATPase activity was U/mg protein.

### 2.9. Circular Dichroism (CD) Spectra

Intracellular protein and nucleic acid structures were determined using CD spectra (Chirascan Plus, Applied Photophysics, Surry, ND, UK) using the intracellular solution described in Section 2.6 (in PBS) in accordance with previous research [32]. The detection wavelengths of the protein and nucleic acid were 200–250 nm and 250–320 nm, respectively. The scanning rate was 50 nm/min, and the width of the cuvette was 1 mm.

### 2.10. Atomic force Microscopy (AFM)

AFM was performed to observe the morphological changes of *E. coli* O157:H7 after different treatments. Bacterial suspensions of the HHP and PCA + HHP groups were prepared as described in Section 2.4 and then fixed with glutaraldehyde fixative overnight. Each mixture was washed three times and resuspended in sterilized water. Each sample was placed onto a mica sheet and naturally dried before observing using a Bruker multimode 8 AFM (Bruker Corporation, Germany) in auto scan mode.

### 2.11. Statistical Analysis

All the experiments were performed in triplicate to confirm data credibility. Data were presented as the mean values ± standard deviations. The differences within groups were processed using a one-way analysis of variance, followed by Duncan’s tests, a method used for comparing the differences between multiple samples. The differences between groups were determined by *t*-tests. The Duncan and *t*-tests were both carried out using SPSS software (version 23; SPSS, Inc., Chicago, IL, USA). *p* ≤ 0.05 was considered statistically significant.

## 3. Results

### 3.1. Determination of the PCA Treatment Conditions

The MIC of the PCA on *E. coli* O157:H7 was 2.5 mg/mL, which was same in Nuding et al. [30]. To ensure that the bacteria did not severely lose vitality after the single PCA treatment, a lower PCA concentration was selected to for the synergetic treatment. As shown in Table 1, when the PCA concentrations were at 1/4 MIC and 1/8 MIC (0.625 and 0.3125 mg/mL, respectively), the decreases in the surviving population were both less than 0.5 log CFU/mL, whereas those at 1/2 MIC and 1MIC (1.25 and 2.5 mg/mL, respectively) were 0.95 and 1.72 log CFU/mL, respectively. Moreover, when treated with 1/2-MIC PCA for 60 min, the sub-lethal injury rate reached 99.99% (data not shown), which indicated that the living bacteria were in a generally injured state [33]. Therefore, the 1/2-MIC PCA concentration was used in the following synergetic bactericidal study. For this concentration, the numbers of surviving cells were not significantly different (*p* = 0.474) after 60- and 75-min treatment times. Consequently, the PCA treatment conditions were determined as 1.25 mg/mL (1/2 MIC) and 60 min.

### 3.2. The Bactericidal Effects of Synergetic Treatments

The surviving *E. coli* O157:H7 populations after no, single PCA, HHP and PCA + HHP treatments are shown in Table 2. Among the different conditions, 0 MPa for the HHP group indicated untreated cells, and 0 MPa for the PCA + HHP group indicated cells treated with only PCA. The initial microbial count of *E. coli* O157:H7 was approximately 9.0 log CFU/mL. As the pressure increased, the surviving colony populations decreased in both the HHP and PCA + HHP groups. In the HHP group, the colony population started to decrease significantly when the pressure reached 300 MPa. When the pressure reached a maximum (500 MPa for 5 min), there were 4.48 log CFU/mL of colonies reduced, which was similar to the findings of Arbol et al. [34], in which the *E. coli* O157 strain was reduced 5.36 log CFU/mL after 500 MPa for 8 min. The number of surviving colonies was reduced considerably by 1.07 log CFU/mL after the single PCA treatment. After the combined PCA + HHP treatment, the surviving colonies kept significantly decreasing with every 100-MPa increase in pressure, which could result from the difference of the pH value in the solution HHP and PCA + HHP treatments, or the PCA treatment affecting the tolerance of the membrane to HHP [35]. When treated with 100 MPa for 5 min, the bactericidal effect of the synergetic treatment (1.53 log CFU/mL reduction) was the same as the sum of the two separate treatments. After the pressure reached 200 MPa, the bactericidal effect of the synergetic treatment (2.38 log CFU/mL reduction) was not only higher than either the single PCA (1.06 log CFU/mL) or 200 MPa (0.52 log CFU/mL) treatment, but also higher than the sum of two separate treatments. The gap between the treatments was positively correlated with the pressure. There was an approximate 9 log CFU/mL bacterial colony reduction in the PCA + HHP group, whereas the sum of the two separate treatments was only 5.54 log CFU/mL when the pressure reached 500 MPa. Thus, the PCA and HHP treatments had synergetic roles in the bactericidal effect when the pressure was greater than 200 MPa.

### 3.3. Determination of Membrane Permeability

**Figure 1:**
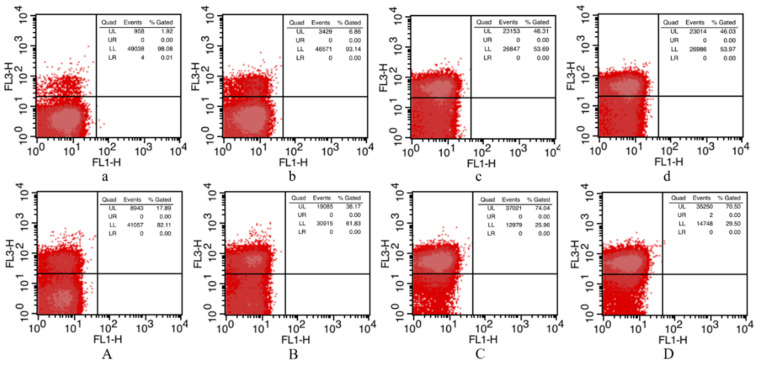
The determination of membrane permeability of sample after different treatments by propidium iodide (PI) uptake method. The results were presented in cross quadrant images. (**a**) and (**A**), untreated sample and sample with single 1/2 MIC PCA treatment (1.25 mg/mL for 60 min at 37 °C), respectively; (**b**) to (**d**) and (**B**) to (**D**), HHP group and PCA + HHP group samples with 100, 300, 500 MPa pressure treatments for 5 min, respectively. Percentage of cells in each quadrant was labelled on the top right-hand corner. UL, upper left region; UR, upper right region; LL, lower left region; LR, lower right region. The vertical axis and abscissa axis represented the relative fluorescence intensity at 488 nm and 530 nm.

PI is a nucleotide-binding probe that produces red fluorescence when crossing damaged membranes, and it is commonly used in the determination of membrane permeability. As shown in Figure 1, the untreated cells were mainly distributed in the lower-left (LL) area (98.08%), which represents intact cells, for which the relative fluorescence intensity at 488 nm (red fluorescence) was low. As the pressure increased, the PI stained cells distributed in the upper-left area increased from 1.92% to 46.03%. Thus, the intensity of the red fluorescence increased, which indicated that the membrane permeability increased along with the high pressure, resulting in more PI going through the membrane and binding with nucleic acid [36]. The single PCA treatment results, shown in Figure 1A, reveal that the membrane permeability had changed, with the stained cells increasing to 17.89%. The same phenomenon in which a PCA treatment of 1/2-MIC concentration increased *Cronobacter sakazakii* membrane permeability was discovered by Jia et al. [20]. After being exposed to PCA + HHP, the stained cells increased to 70.50%, which indicated that damage to the membrane permeability of the PCA + HHP group was greater than the combined damage caused by the two treatments independently (63.92%). When treated with 500 MPa, the numbers of stained cells remained almost unchanged, or slightly decreased, compared with 300 MPa in both the HHP and PCA + HHP groups.

### 3.4. Determination of the Intracellular Constituent Contents

**Figure 2:**
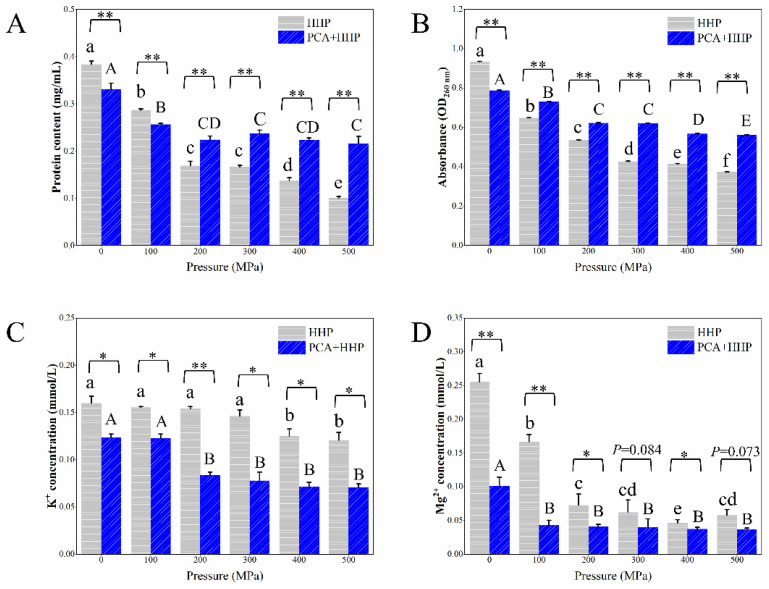
The effect on the amounts of intracellular protein (**A**), nucleic acid (**B**), K^+^ (**C**) and Mg^2+^ (**D**) in *E. coli* O157:H7 after different treatments. Different letters a–f and A–E mean differ significantly within the HHP group and PCA + HHP group (*p* ≤ 0.05, Duncan), respectively. Symbol * and ** mean the difference is significant at the 0.05 and 0.01 level, respectively, between the HHP group and PCA + HHP group (*t*-test). The HHP group was *E. coli* O157:H7 treated with 0 (untreated cells), 100, 200, 300, 400 and 500 MPa for 5 min, respectively. The PCA + HHP group was *E. coli* O157:H7 treated with 1/2 MIC PCA treatment (1.25 mg/mL for 60 min at 37 °C) combined with 0 (single PCA treated cells), 100, 200, 300, 400 and 500 MPa for 5 min, respectively.

The intracellular constituent contents caused by different treatments are shown in Figure 2. The intracellular protein content in the untreated cells was 0.38 mg/mL, which decreased to 0.10 mg/mL (500 MPa for 5 min) as the pressure increased in the HHP group. When treated with PCA only, the protein content slightly decreased to 0.33 mg/mL. However, the protein content in the PCA + HHP group was significantly (*p* ≤ 0.01) higher than that in the HHP group at pressures greater than 200 MPa. Additionally, the protein content showed no significant change after the pressure reached 300 MPa, nor did the intracellular nucleic acid contents (Figure 2B). The intracellular nucleic acid content of the HHP group was significantly lower than that of the PCA + HHP group. The intracellular Mg^2+^ and K^+^ contents are the most and second-most abundant divalent cations in living cells, respectively [37]. As shown in Figure 2C, the intracellular K^+^ content decreased (from 0.16 to 0.12 mmol/L) as the pressure increased. Compared with the HHP group, the intracellular K^+^ content was lower in the PCA + HHP group. The intracellular Mg^2+^ content (Figure 2D) showed the same trend as that of K^+^. Significantly, the Mg^2+^ concentration decreased to 0.10 mmol/L, which was less than half that of the untreated cells, after the PCA only treatment. The decrease of the intracellular content of K+ and Mg2+ may result from the ion leakage, which is related to the changes in membrane permeability and integrity [32,38]. In addition, the transport of Mg^2+^ and K^+^ is related to corresponding transmembrane transporters, which could be inhibited by low pH [39,40]. Thus, the continuous decreases in the Mg^2+^ and K^+^ concentrations might be caused by changes in membrane permeability and the inhibition of transporters.

### 3.5. Determination of Key Enzyme Activities

**Figure 3:**
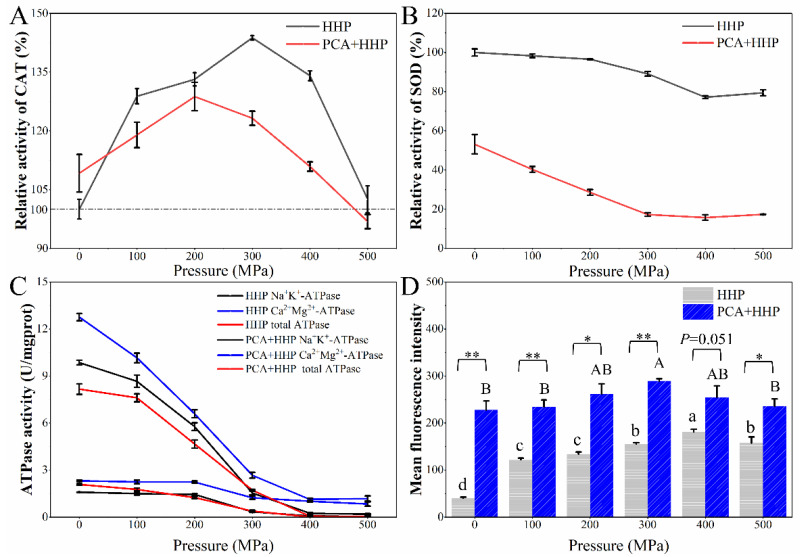
The relative activity of CAT (**A**), SOD (**B**), ATPase (**C**, Na^+^ K^+^-ATPase, Ca^2+^ Mg^2+^-ATPase and total- ATPase activity) and the change of membrane potential (**D**) of *E. coli* O157:H7 after different treatments. Different letters a–d and A,B mean differ significantly within the HHP group and PCA + HHP group (*p* ≤ 0.05, Duncan), respectively. * *p* ≤ 0.05 and ** *p* ≤ 0.01 mean differ significantly within HHP and PCA + HHP group.

**Figure 4:**
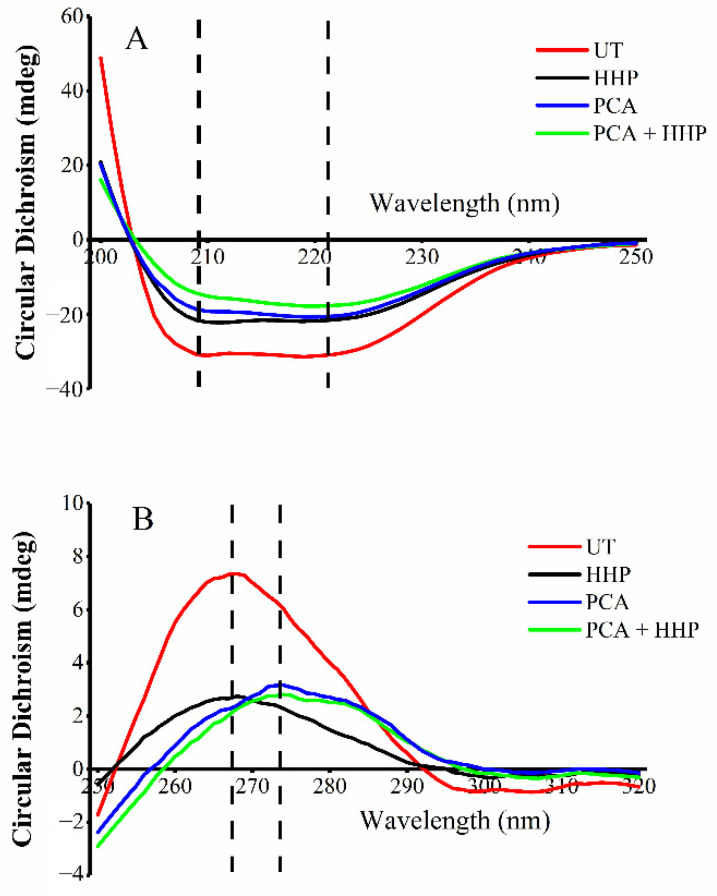
Circular dichroism of intracellular protein (**A**) and nucleic acid (**B**) after different treatments. UT (red line), untreated control; HHP (black line), sample with 300 MPa pressure for 5 min treatment; PCA (blue line), sample with 1/2 MIC PCA treatment (1.25 mg/mL for 60 min at 37 °C); PCA + HHP (green line), sample treated with 300 MPa pressure combined with 1/2 MIC PCA treatment.

The CAT activity patterns, as shown in Figure 3A, in the HHP and PCA + HHP groups revealed a similar trend of increasing first and then decreasing as the pressure increased. In the HHP group, the CAT activity increased by 50% when the pressure reached 300 MPa and then decreased to 102.00%. When treated with PCA only, the CAT activity significantly increased to 109.18% (*p* ≤ 0.05). Shi et al. [23] found that at a molecular level that gene *katG*, which encodes KatG enzyme (a distinct CAT), is up-regulated in *E. coli* after a lactic acid treatment. After the PCA + HHP treatment, the CAT activity reached a maximum (128.76%) when the pressure reached 200 MPa, and then it decreased to 96.85%. As shown in Figure 3B, SOD activity was negatively correlated with pressure in both the HHP and PCA + HHP groups. Without the PCA treatment, the SOD activity decreased only 21% when the pressure reached 500 MPa (Figure 4A). However, the SOD activity decreased to 53.13% after the PCA only treatment and declined steadily until the pressure reached 300 MPa. Identical results were obtained by Kang et al. [41,42] in which the SOD activity was inhibited by a citric acid (CA) only treatment, and it decreased more when CA was combined with a hot water treatment.

Ca^2+^Mg^2+^-ATPase and Na^+^K^+^-ATPase catalyze the hydrolysis of ATP that is coupled to the active transport of Ca^2+^/Mg^2+^ and Na^+^/K^+^, respectively, across the cell membrane, which play essential roles in the transduction of signals, maintenance of cell homeostasis and decomposition and synthesis of ATP [42,43]. The activities of Na^+^K^+^-ATPase, Ca^2+^Mg^2+^-ATPase and total-ATPase are shown in Figure 3C. In the HHP group, the ATPase activities decreased as the pressure increased, which indicated that ATPase could be inactivated by high pressure. Similarly, Gao et al. [44] found that a decrease in the ATPase activity by HHP led to a more significant inactivation of *Listeria monocytogenes*. In the PCA + HHP group, the Na^+^K^+^-ATPase, Ca^2+^Mg^2+^-ATPase and total-ATPase activities rapidly decreased to approximately 1/6, 1/5 and 1/4 of the initial concentrations after the PCA only pre-treatment, respectively. They then decreased slightly as the pressure increased.

### 3.6. Determination of Membrane Potential

The membrane potential was determined using DiBAC(4)_3_, an anionic lipophilic dye that enters only depolarized cells and then fluoresces by binding reversibly to the hydrophobic core of the lipid membrane [45]. The mean fluorescence intensities (MFIs) are enhanced when the dye enters the cells owing to membrane depolarization [46]. As shown in Figure 3D, the MFIs of both the HHP and PCA + HHP groups increased first and then decreased, and the turning points were 400 and 300 MPa, respectively. Furthermore, the MFI of the PCA + HHP group was obviously higher than that of the HHP group. The MFI of the sample treated with only PCA was nearly six-fold that of the untreated sample, which indicated that the PCA treatment significantly increased membrane depolarization.

### 3.7. Determination of Intracellular Protein and Nucleic Acid Structures

The structures of intracellular proteins and nucleic acids were determined using CD spectroscopy. As shown in Figure 4A, two negative peaks at approximately 210 nm and 220 nm were observed, which corresponded to the absorptions of α-helices [47]. These two negative peaks became smoother after the HHP, PCA and synergetic treatments, which indicated decreasing of α-helices contents. Among the treatments, the synergetic treatment had the greatest effect on protein secondary structures.

The CD spectroscopy of the nucleic acid structure is shown in Figure 4B. The positive peak position in HHP group revealed almost no change, demonstrating that HHP only resulted in loose base stacking and double-helix structures, which was similar to the results of Zhu et al. [32]. Unlike the HHP treatment, the positive peaks of the nucleic acid structures in the PCA and PCA + HHP groups redshifted slightly to the same site. Thus, we inferred that the PCA treatment caused the change in the DNA configuration.

### 3.8. Morphological Changes

**Figure 5:**
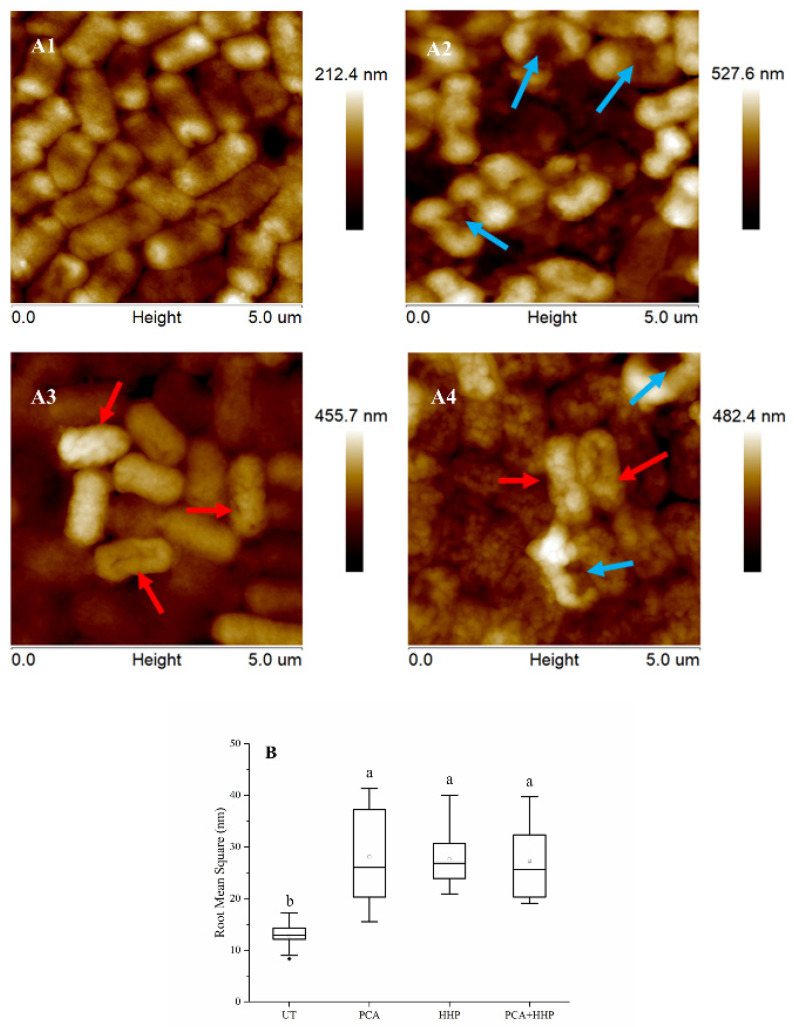
Morphological changes of *E. coli* O157:H7 caused by different treatments. A1, A2, A3 and A4 were atomic force microscopy (AFM) height images of untreated group, HHP group, PCA group and PCA + HHP group, respectively. Red arrowheads indicated cells with shrunken surface and blue arrowheads holes or depressions. (**B**) was the average bacterial surface RMS in nm obtained on 605.1 × 605.1 nm^2^ areas of surfaces of cells with different treatments. For each group, 10 cells were plotted, respectively. a–b mean statistically significant between different groups (*p* ≤ 0.05, Duncan).

The AFM height images of *E. coli* O157:H7 cells captured in water using the tapping mode are shown in Figure 5A. The untreated cells (A1) were uniformly distributed and presented an intact and smooth membrane. After the HHP treatment (300 MPa for 5 min), holes and depressions on the cell surfaces were apparent. When exposed to PCA only, the cell surface shrunk significantly. For the PCA + HHP group, there were not only depressions on the membrane but the surfaces had also shrunken, which corresponded to the combination of the two separate treatments. Thus, we inferred that the synergetic PCA + HHP treatment resulted in more severe morphological injuries than either single treatment. A similar result was obtained by Huu et al. [48], in which increased *E. coli* O157:H7 membrane damage observed by SEM was induced by the combination of low-intensity ultrasound and propyl gallate. In addition to the morphological observations, the roughness of the cell membrane is an important cytological parameter mainly obtained using the AFM technique and it is also a sensitive indicator of the cell’s health [49]. As shown in Figure 5B, greater roughness values, 2.19-, 2.16- and 2.13-fold more than that of untreated cells, were found after PCA, HHP and PCA + HHP treatments, respectively.

## 4. Discussion

This research studied the bactericidal effects and mechanisms of a new decontamination method, which combined HHP technology with PCA. According to our results, the combination of HHP and PCA produced a synergetic bactericidal effect when the pressure was greater than 200 MPa. When the pressure reached 500 MPa, the number of bacterial colonies was reduced by approximately 9.0 log CFU/mL (Table 2). According to Montiel et al. [50], the combination of HHP (450 MPa for 5 min) and reuterin (16 mmol/L), lactoperoxidase system (2.8 U/g) or lactoferrin (1 g/L) treatments of cold-smoked salmon results in a 1.8–3.5 log CFU/mL reduction. In addition, Bulut and Karatzas [51] found that the combination of HHP (441 MPa for 5 min) and freezing, at 4 °C and −24 °C, leads to 1.01 and 5.19 log CFU/mL, respectively, which were lower than in our research. Thus, it could be inferred that this new method has a superior bactericidal effect. The colony population did not decrease significantly until 300 MPa in the HHP group. However, this phenomenon was not found in the PCA + HHP group; consequently, we inferred that the PCA + HHP combination might act not only on the membrane but also on other sites.

The impacts of HHP and PCA + HHP on membranes were assessed by investigating membrane permeability, intracellular constituent contents, membrane potentials and morphological changes. The PCA treatment itself increased membrane permeability, perhaps because the hydrophobic -OH structure of PCA bound to the lipid bilayer of the membrane, thereby changing the membrane’s structure [52]. Notably, the numbers of stained cells at 500 MPa remained almost unchanged, or decreased slightly, in both the HHP and PCA + HHP groups compared with that at 300 MPa, which was inconsistent with the previous bactericidal effect results (Figure 1 and Table 2). Pagan et al. [53] found that membranes are unable to reseal after 300-MPa treatments, but could reseal after higher pressure treatments. Reitermayer et al. [54] proposed that membrane permeabilization, which has frequently been postulated as a major factor in the HHP-driven inactivation of microbes, is not necessarily required for HHP-induced cell death. Thus, membrane permeability no longer increases after 300 MPa.

Cell contents do not leak unless the membrane integrity and permeability have been altered [55]. As shown in Figure 2, the intracellular protein, nucleic acid, K^+^ and Mg^2+^ contents all showed downward trends, indicating that the leakage of intracellular constituents was induced by the increase in membrane permeability. However, the protein and nucleic acid leakage in the PCA + HHP group was less than that of HHP group (Figure 2A,B), which contradicted with PI-based results, in which the PCA + HHP treatment led to greater membrane permeability. According to Shi et al. [27], a slight increase in permeability may lead to K^+^ and Mg^2+^ leakage, but protein and nucleic acid leakage would require severe membrane damage (such as holes in the membranes, which were found in the AFM results). The K^+^ and Mg^2+^ leakage levels, of the PCA + HHP group, were higher than those of the HHP group (Figure 2C,D). The PI passed through the damaged membrane, but passage was not correlated with the degree of damage. Thus, it may be speculated that the degree of damage in the HHP group was more severe than that of the PCA + HHP group. Kim and Robey also found that the acid resistance system gene *ropS* in *E. coli* O157:H7, which also positively regulates the stress tolerance of cells, is up-regulated after exposure to a low-pH environment [56,57]. In summary, after the PCA treatment, the tolerance of cells to HHP was indeed increased. However, the synergetic PCA + HHP treatment produced a greater bactericidal effect, which might be because the PCA treatment also acted on other important sites except membranes.

The membrane potential may regulate the ion transport across the membrane and the intracellular pH level, which is important for the maintenance of cell homeostasis [58]. Kong et al. [59] obtained a similar result in which salicylic acid (also a kind of benzoic acid) significantly influenced the membrane potential. The decrease after the turning point may be due to the massive death of *E. coli* O157:H7, which was consistent with our previous results (Table 2). Hossain et al. [60] found that membrane potential plays a vital role in local ruptures of lipid bilayers that induce rapid permeabilization, resulting in bactericidal activity. Thus, the enhancement of membrane depolarization may be the reason that the synergetic treatment had a better antibacterial effect. The AFM results revealed that HHP mainly induced the formation of holes and PCA induced shrinkage. As shown in Table 1, the bactericidal effect of the PCA + HHP group was more significant than those of the individual PCA and HHP groups. However, there were no significant differences among the roughness values of these groups (Figure 5B). Previous research [61] indicated that roughness values are associated with cell shrinkage, whereas HHP treatments mainly lead to surface holes. This may be why there were no significant changes in roughness values among the different treatments.

Inside the cells, the enzyme activities and protein and nucleic acid structures were determined. CAT and SOD are two essential enzymes in the defense system against intracellular oxidative stress. Bravim et al. [62] found that HHP treatments cause the generation of reactive oxygen species (ROS) inside cells. Aertsen et al. [63] further verified that oxidative bursts are more prominent and, therefore, destructive in active cells. Thus, the increase in CAT activity may be the cellular response to avoid damage caused by ROS accumulation. The response level of the PCA + HHP group was lower than that of the HHP group, which may be explained by the greater damage caused by the synergetic bactericidal treatment (Figure 3A). Inaoka et al. [64] indicated that the gene encoding SOD is more sensitive in resisting oxidative stress caused by HHP compared with CAT in *Bacillus subtilis*, which could explain why the SOD and CAT trends differed (Figure 3B). The ATPase activity showed a downward trend (Figure 3C), and the inhibition of the acid treatment was particularly significant. Similar results were obtained by Jayaram et al., [65] in which PCA reduces the gastric H^+^, K^+^-ATPase enzyme activity. In Yin’s research [66], PCA also significantly lowered the Na^+^K^+^-ATPase activity in five cancer cell lines. The ATPase is located at the cell membrane, and the PCA and HHP treatments both damage membranes, including the membrane components, membrane permeability and integrity [52,67]. This may have significantly inhibited the ATPase activity.

Based on CD spectral results, changes in protein secondary structures were mainly expressed by reductions in the α-helix (Figure 4A). Proteins are misfolded and aggregated after the HHP treatment, which might lead to cytotoxicity and cell membrane destruction [68,69]. Additionally, when a protein encounters a low-pH environment, the protein structure might be destroyed, and the PCA treatment could induce an acidic environment [70]. These results may explain the changes in protein structures caused by the synergetic treatment. The nucleic acid changes were mainly expressed by the redshift of the absorbance peak (Figure 4B). Bacterial DNA is severely broken and degraded by eugenol [71]. Additionally, Cui et al. [72] observed that a vine tea aqueous extract binds with DNA by intercalation and groove binding, which is expressed as the redshift of the UV spectroscopy. Thus, the PCA treatment might cause changes in the nucleic acid form or combine with DNA, and the synergetic treatment may cause more significant changes, which lead to nucleic acid structure changes and cell death.

## 5. Conclusions

Against *E. coli* O157: H7, the PCA + HHP treatment displayed a synergetic bactericidal effect when the pressure was greater than 200 MPa. The mechanisms of synergetic sterilization were mainly related to changes in membrane permeability, intracellular and extracellular enzyme activities (SOD, CAT and ATPase), intracellular macromolecule structures, morphology and intracellular substances contents (protein, nucleic acid and ions). More specifically, the HHP treatment mainly destroyed the cell membrane, whereas the combined PCA + HHP treatment also significantly affected nucleic acid structures and membrane-associated enzyme activities. The results that suggested the synergetic PCA + HHP treatment could be used as a new bactericide owing to the treatment’s good bactericidal effects and multiple targets. In addition, PCA has the potential to become a food additive owing to its multiple targets.

## Figures and Tables

**Table 1:** The surviving population (log colony forming units per milliliter, CFU/mL) of *E. coli* O157:H7 after single protocatechuic acid (PCA) treatments with different concentrations (1/8, 1/4, 1/2, 3/4, 1 minimum inhibitory concentration, MIC) and treatment times (0, 15, 30, 45, 60, 75 min).

	1/8 MIC	1/4 MIC	1/2 MIC	1 MIC
0 min	9.15 ± 0.00 ^a,A^	9.19 ± 0.10 ^a,A^	9.14 ± 0.02 ^a,A^	9.15 ± 0.00 ^a,A^
15 min	9.10 ± 0.08 ^a,A^	9.14 ± 0.02 ^a,A^	9.07 ± 0.08 ^a,A^	8.95 ± 0.04 ^b,B^
30 min	9.07 ± 0.04 ^ab,A^	9.08 ± 0.02 ^a,A^	8.89 ± 0.22 ^a,A^	8.40 ± 0.10 ^c,B^
45 min	8.99 ± 0.07 ^bc,A^	8.92 ± 0.10 ^b,A^	8.48 ± 0.07 ^b,B^	7.84 ± 0.00 ^d,C^
60 min	8.97 ± 0.01 ^bc,A^	8.88 ± 0.07 ^b,A^	8.32 ± 0.18 ^bc,B^	7.58 ± 0.04 ^e,C^
75 min	8.95 ± 0.07 ^c,A^	8.80 ± 0.06 ^b,A^	8.20 ± 0.11 ^c,B^	7.43 ± 0.03 ^f,C^

1. Values are means ± standard deviations from three replications. 2. Different letters a–f and A–C mean differ significantly within the same PCA concentration and the same processing time, respectively (*p* ≤ 0.05, Duncan test). 3. The 1/8, 1/4, 1/2 and 1 MIC of PCA against *E. coli* O157:H7 was 0.3125, 0.625, 1.25 and 2.5 mg/mL, respectively.

**Table 2:** The surviving populations (log CFU/mL) of *E. coli* O157:H7 after the high hydrostatic pressure (HHP) treatment (HHP group) and the synergetic treatment of PCA (1.25 mg/mL for 60 min at 37 °C) and HHP (PCA + HHP group).

Treatments	HHP Group	PCA + HHP Group
0 MPa	9.08 ± 0.04 ^a^	8.02 ± 0.06 ^A^
100 MPa, 5 min	8.61 ± 0.61 ^a,^*	7.55 ± 0.14 ^B^
200 MPa, 5 min	8.56 ± 0.02 ^a,^**	6.70 ± 0.36 ^C^
300 MPa, 5 min	7.53 ± 0.09 ^b,^**	5.66 ± 0.05 ^D^
400 MPa, 5 min	6.59 ± 0.04 ^c,^**	3.45 ± 0.12 ^E^
500 MPa, 5 min	4.60 ± 0.20 ^d,^**	N.D.

1. N.D. means not detected (detection limit: 1 log CFU/mL). 2. Values are means ± standard deviations from three replications. 3. Different letters a-d and A–E mean differ significantly within the HHP group and PCA + HHP group, respectively (*p* ≤ 0.05, Duncan test). 4. Symbol * and ** mean the difference is significant at the 0.05 and 0.01 level, respectively, between the HHP group and PCA + HHP group (*t*-test).

## Data Availability

Not applicable.
